# A Novel Inflammatory-Nutritional Prognostic Scoring System for Stage III Gastric Cancer Patients With Radical Gastrectomy Followed by Adjuvant Chemotherapy

**DOI:** 10.3389/fonc.2021.650562

**Published:** 2021-06-14

**Authors:** Nan Wang, Wenqi Xi, Sheng Lu, Jinling Jiang, Chao Wang, Zhenglun Zhu, Chao Yan, Jing Liu, Jun Zhang

**Affiliations:** ^1^ Department of Oncology, Ruijin Hospital, Shanghai Jiao Tong University School of Medicine, Shanghai, China; ^2^ Department of General Surgery, Ruijin Hospital, Shanghai Jiao Tong University School of Medicine, Shanghai, China; ^3^ State Key Laboratory of Oncogenes and Related Genes, Shanghai Jiao Tong University School of Medicine, Shanghai, China

**Keywords:** gastric cancer, prognosis, immunonutritional status, nomogram, scoring system

## Abstract

**Purpose:**

The present study was designed to explore the prognostic value of preoperative inflammatory and nutritional biomarkers in stage III gastric cancer (GC) patients with adjuvant chemotherapy and to develop a novel scoring system called the inflammatory-nutritional prognostic score (INPS).

**Methods:**

A total of 513 patients with pathological stage III GC undergoing radical gastrectomy followed by adjuvant chemotherapy from 2010 to 2017 were enrolled in the study. Clinicopathological characteristics and blood test parameters of individual patients were collected. The least absolute shrinkage and selection operator (LASSO) Cox regression model was used for feature selection to construct INPS. Survival curves were generated using the Kaplan-Meier method with log-rank tests. The nomogram was generated based on the result of the multivariate analysis using Cox’s proportional hazards model. The model was assessed by the concordance index (C-index) and was internally validated by bootstraps.

**Results:**

According to the results of Lasso Cox regression and K-M survival curves, INPS was determined as follows: a low body mass index (BMI) (<23 kg/m^2^), a low prealbumin (<180 mg/L), a high neutrophil-lymphocyte ratio (NLR) (≥2.7), a high platelet-lymphocyte ratio (PLR) (≥209.4), a low lymphocyte-monocyte ratio (LMR) (<2.8), and a low prognostic nutritional index (PNI) (<45.1); each were scored as 1, and the remaining values were scored as 0. The individual scores were then summed up to construct the INPS and further divided into 4 groups: Low Risk (INPS 0); Low-medium Risk (INPS 1); High-medium Risk (INPS 2-4); and High Risk (INPS 5-6). In multivariate analysis, INPS was an independent predictor of overall survival (OS) in stage III GC, with the 5-year OS rates of 70.8%, 57.4%, 41.5%, and 30.6%, respectively. The nomogram based on INPS and other independent predictors (gender, pT stage, pN stage, lymphovascular invasion, and CEA level) showed good predicting performance with a C-index of 0.707, which was superior to the TNM stage alone (C-index 0.645, *p*=0.008) and was internally validated with the corrected C-index of 0.693.

**Conclusion:**

Preoperative INPS was an independent prognostic factor of stage III GC patients with radical surgery followed by adjuvant chemotherapy. The nomogram based on INPS may serve as a simple and potential model in risk stratification and guiding treatment strategies in clinical practice.

## Introduction

In China, gastric cancer (GC) is the second most common cancer and the third leading cause of death among all malignancies, accounting for 45% of GC-related mortality worldwide (1, 2). Due to the lack of effective screening, the majority of GC patients in China are diagnosed at an advanced stage, and the overall 5-year survival rate is less than 40% (3). Especially for stage III GC, even with radical surgery and standard adjuvant chemotherapy, the prognosis is still very poor and highly heterogeneous (4–6). Thus, it is necessary to identify a more accurate and cost-effective prognostic model for individualized risk stratification and optimal therapeutic strategies for stage III GC patients.

In recent years, increasing evidence indicates that there is a specific triangle connection between nutrition, inflammation, immunity, and cancer (7). Preoperative biomarkers in peripheral blood, to some extent, reflecting the baseline nutritional and immune status of patients are considered as potential markers for predicting the prognosis, due to high accessibility in clinical practice (8–10). In GC, several studies have reported some inflammation-based indexes, such as the neutrophil-lymphocyte ratio (NLR), lymphocyte-monocyte ratio (LMR), platelet-lymphocyte ratio (PLR), and immune inflammation index (SII), and nutrition-based parameters, such as the body mass index (BMI), albumin, and prognostic nutritional index (PNI), are associated with the prognosis (11–13). Some studies combined known prognostic factors to establish a new scoring system to predict prognosis and guide clinical practice. For instance, Gao et al. reported that TNM-PNI was a new and effective prognostic indicator for patients with GC after curative D2 resection (14). However, so far, no studies have comprehensively integrated these inflammatory, nutritional, and clinicopathological parameters to predict the prognosis, especially for stage III GC patients. We believe that integrating these markers might provide more accurate prognostic information and is more meaningful than individual indicators.

Therefore, we designed this retrospective study and aimed to explore a novel prognostic scoring system, which we called the inflammatory-nutritional prognostic score (INPS), based on preoperative inflammatory and nutritional biomarkers integrating with clinicopathologic parameters, to predict outcomes in stage III GC patients undergoing curative gastrectomy followed by adjuvant chemotherapy.

## Materials and Methods

### Patients and Study Design

A total of 513 GC patients undergoing curative gastrectomy followed by adjuvant chemotherapy at Ruijin Hospital Affiliated with Shanghai Jiao Tong University School of Medicine between February 2010 and October 2017 were enrolled in this study. Inclusion criteria included the following: 1) R0 resection with D2 lymphadenectomy; 2) stage pIII (according to UICC/AJCC cancer staging 8th edition) gastric adenocarcinoma confirmed by postoperative histopathology; 3) completed adjuvant chemotherapy after surgery unless disease progression or death; 4) no preoperative chemotherapy or radiotherapy; 5) no preoperative parenteral nutrition, acute inflammation, or other immune diseases; 6) no history of other malignancies; and 7) intact clinicopathologic and follow-up data. Patients who underwent emergency surgery due to bleeding, perforation, or obstruction or those that died of operative complications within 30 days after surgery were excluded. Adjuvant chemotherapy regimen included 5-fluorouracil based (5-FU) combinational chemotherapy and mono-chemotherapy.

### Data Collection and Definition of Variables

Clinicopathological information of individual patients was collected, including gender, age at diagnosis, tumor sites, histological grade, pathological tumor type, pTNM stage (AJCC cancer staging 8th edition), lymphovascular/perineural invasion, and adjuvant chemotherapy regimen. Preoperative body mass index (BMI) and blood tests were done within 2 weeks before surgery. The continuous variables (normal value), including CA12-5 (35 U/ml), CA19-9 (35 U/ml), CEA (5 ng/ml), AFP (8.78 ng/ml), hemoglobin (120 g/L), total protein (60 g/L), prealbumin (180 mg/L), albumin (35 g/L), albumin-globulin ratio (AGR, 1.25), neutrophil count (7×10^9^/L), lymphocyte count (0.8×10^9^/L), monocyte count (1×10^9^/L), and platelet count (320×10^9^/L), were grouped according to the standards developed by the clinical laboratory of Ruijin Hospital. The optimal cut-off of preoperative BMI(23 kg/m^2^) was determined by Asian-specific criteria based on our previous study (15).

The neutrophil-lymphocyte ratio (NLR), platelet-lymphocyte ratio (PLR), lymphocyte- monocyte ratio (LMR), systemic immune inflammation index (SII), and prognostic nutritional index (PNI) were calculated as follows: NLR= N/L, PLR=P/L, LMR=L/M, SII= P×N/L, and PNI= albumin (g/L) + 5×L (10^9^/L) (N: neutrophil count, P: platelet count, L: lymphocyte count, and M: monocyte count). The optimal cut-off levels of these markers were determined using the R package “maxstat” (Maximally Selected Rank Statistics) based on overall survival (OS) in the present study.

### Patients Follow-Up

Follow-up assessment included physical examinations and blood tests, including tumor markers and imaging examinations (chest, abdomen, and pelvic CT or MRI scan with contrast) every 3 months in the first 2 years and then every 6 months until 5 years after surgery. Endoscopy was performed annually. The latest follow-up date was December 2019, with a median follow-up of 59.5 months (95% CI: 55.5-63.5). OS was defined as the time from primary surgery until death from any cause.

### Statistical Analysis

Continuous and categorical variables were summarized using medians [interquartile ranges (IQRs)] and frequencies (percentages). A Chi-square test or Fisher’s exact test was used to test the association between categorical variables. Due to the presence of multicollinearity, we used the least absolute shrinkage and selection operator (LASSO) Cox regression model for dimensionality reduction to select the most useful prognostic features out of all the available inflammatory and nutritional biomarkers. The method uses an L1 penalty to shrink some regression coefficients to exactly zero. To find an optimal λ, 10-fold cross-validation with minimum criteria was employed. The retained features with nonzero coefficients were used to establish a novel inflammatory-nutritional prognostic score (INPS). Survival curves were generated using the Kaplan-Meier method with log-rank tests. To identify the independent prognostic factors, univariate and multivariate analyses were performed using Cox’s proportional hazards model. The variables with a *p*-value of 0.1 or less in univariate analysis were included in the multivariate analysis. Finally, a prognostic nomogram was generated based on the result of multivariate analysis, with the discriminative ability assessed by the concordance index (C-index) and the goodness of fit assessed by R^2^. Calibration curves were performed to compare the predicted probability of OS with the actual outcome. The model was internally validated by bootstraps with 1,000 resample. A two-tailed p-value of less than 0.05 was considered to be statistically significant. Statistical analyses were carried out using SPSS version 24.0 and R language version 4.0.2 (http://www.R-project.org).

## Results

### Patients’ Characteristics and INPS Construction

The baseline clinicopathologic characteristics are shown in **Table 1**. Of the 513 enrolled patients, 356(69.4%) were male and 157(30.6%) were female; 219(42.7%) were stage IIIA, 189(36.8%) were stage IIIB and 105(20.5%) were stage IIIC. The median age at diagnosis of GC was 60 years old (IQR: 53~67). During the follow-up period, 278(54.2%) patients were detected as local recurrence or distant metastasis. Among them, 243(47.4%) patients died. The 5-year OS rates of stage IIIA, IIIB, IIIC patients were 69.5%, 41.0%, and 27.6%, respectively. The baseline information of 15 nutritional and Inflammatory biomarkers is also listed in **Table 1**.

**Table 1:** The baseline clinicopathological characteristics of 513 GC patients.

Patients characteristics	No. of patients (%)	Nutritional and Inflammatory biomarkers	No. of patients (%)
**Gender**		**BMI(kg/m^2^)**	
Male	356(69.4%)	< 23	257(50.1%)
female	157(30.6%)	≥ 23	256(49.9%)
**Age at diagnosis yr.**		**Hemoglobin(g/L)**	
≤ 60	261(50.9%)	< 120	188(36.6%)
> 60	252(49.1%)	≥ 120	325(63.4%)
**Tumor site**		**Total Protein(g/L)**	
Cardia/fundus	79(15.4%)	< 60	110(21.4%)
Body/angulus	184(35.9%)	≥ 60	403(78.6%)
Antrum/pylorus	250(48.7%)	**Prealbumin(mg/L)**	
**Histological grade**		< 180	115(22.4%)
G1/G2	176(34.3%)	≥ 180	398(77.6%)
G3	337(65.7%)	**Albumin(g/L)**	
**Pathological tumor type**		< 35	126(24.6%)
Adenocarcinoma^a^	443(86.4%)	≥ 35	387(75.4%)
Mucinous adenocarcinoma or signet-ring cell carcinoma	70(13.6%)	**AGR**	
**pT stage**		< 1.25	144(28.1%)
T1-3	81(15.8%)	≥ 1.25	369(71.9%)
T4	432(84.2%)	**Neutrophil count(×10^9^/L)**	
**pN stage**		< 7	499(97.3%)
N1	77(15%)	≥ 7	14(2.7%)
N2	128(25%)	**Lymphocyte count(×10^9^/L)**	
N3a	196(38.2%)	< 0.8	11(2.1%)
N3b	112(21.8%)	≥ 0.8	502(97.9%)
**pTNM stage**		**Monocyte count(×10^9^/L)**	
IIIA	219(42.7%)	< 1	509(99.2%)
IIIB	189(36.8%)	≥ 1	4(0.8%)
IIIC	105(20.5%)	**Platelet count(×10^9^/L)**	
**Lymphovascular invasion**		< 320	460(89.7%)
No	276(53.8%)	≥ 320	53(10.3%)
Yes	237(46.2%)	**NLR**	
**Perineural invasion**		< 2.7	342(66.7%)
No	240(46.8%)	≥ 2.7	171(33.3%)
Yes	273(53.2%)	**PLR**	
**Adjuvant chemotherapy**		< 209.4	425(82.8%)
Mono-chemotherapy	81(15.8%)	≥ 209.4	88(17.2%)
Combinational chemotherapy	432(84.2%)		
**CA12-5**		**LMR**	
Normal	470(91.6%)	< 2.8	117(22.8%)
Elevated	43(8.4%)	≥ 2.8	396(77.2%)
**CA19-9**		**SII**	
Normal	392(76.4%)	< 936.7	441(86%)
Elevated	121(23.6%)	≥ 936.7	72(14%)
**CEA**		**PNI**	
Normal	400(78%)	< 45.1	243(47.4%)
Elevated	113(22%)	≥ 45.1	270(52.6%)
**AFP**			
Normal	479(93.4%)		
Elevated	34(6.6%)		

a including papillary, tubular, or mixed adenocarcinoma.

GC, gastric cancer; CA, carbohydrate antigen; CEA, carcinoembryonic antigen; AFP, alpha fetoprotein; BMI, body mass index; AGR, albumin-globulin ratio; NLR, neutrophil-lymphocyte ratio; PLR, platelet-lymphocyte ratio; LMR, lymphocyte-monocyte ratio; SII, systemic immune inflammation index; PNI, prognostic nutritional index.

**Figure 1:**
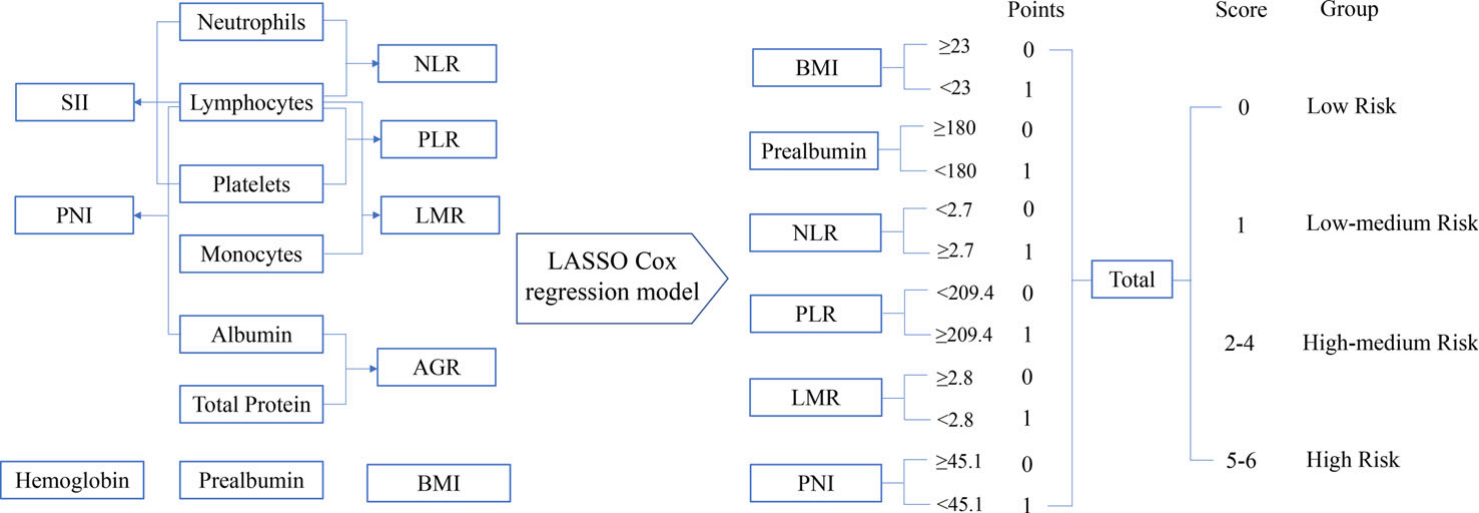
The process diagram of INPS construction and risk stratification. NLR, neutrophil-lymphocyte ratio=N/L; PLR, platelet-lymphocyte ratio=P/L; LMR, lymphocyte-monocyte ratio=L/M; SII, systemic immune inflammation index=P×N/L; PNI, prognostic nutritional index=albumin (g/L)+5×L (10^9^/L) (P, platelet count; N, neutrophil count; L, lymphocyte count; M, monocyte count); AGR, albumin-globulin ratio=albumin/(total protein-albumin); BMI, body mass index=weight(kg)/height(m)^2^; INPS, inflammatory-nutritional prognostic score.

**Figure 2:**
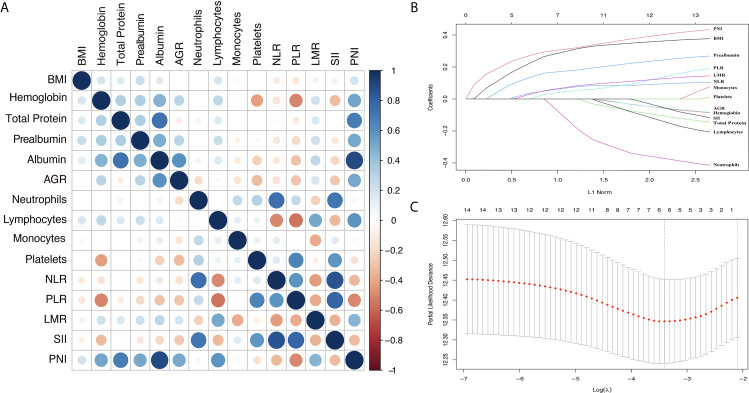
Construction of the INPS using LASSO Cox regression model. **(A)** A correlation matrix is represented from R=-1 negative correlation (red) to R=1 positive correlation (blue). **(B)** LASSO coefficient profiles of the 15 nutritional and Inflammatory biomarkers. **(C)** Ten-fold cross‐validation for tuning parameter selection in the LASSO model. The dotted vertical lines were drawn at the optimal values by minimum criteria and 1-SE criteria. Solid vertical lines represented partial likelihood deviance ± SE. Herein, a value λ = 0.033 with log (λ) = -3.411 was chosen *via* minimum criteria. INPS, inflammatory-nutritional prognostic score; SE, standard error; BMI, body mass index; AGR, albumin-globulin ratio; NLR, neutrophil-lymphocyte ratio; PLR, platelet-lymphocyte ratio; LMR, lymphocyte-monocyte ratio; SII, systemic immune inflammation index; PNI, prognostic nutritional index.

The process diagram of INPS construction and risk stratification is shown in **Figure 1**. The correlation matrix between 15 biomarkers (correlation coefficient R from -1 to1) is represented in **Figure 2A**. Using the LASSO Cox regression model, six features with non-zero coefficients including BMI, prealbumin, NLR, PLR, LMR, and PNI were selected out of all 15 parameters, which corresponded to the optimal value λ.min = 0.033 (**Figures 2B, C**). The novel inflammatory-nutritional prognostic score (INPS) was determined as follows: low BMI (<23 kg/m^2^), low prealbumin (<180 mg/L), high NLR (≥2.7), high PLR (≥209.4), low LMR (<2.8), and low PNI (<45.1) were scored as 1, and the rest of the values were scored as 0. The individual scores were then summed up to construct the INPS, ranging from 0 to 6. According to the survival curves *via* Kaplan-Meier analysis and log-rank tests, the prognosis between INPS 1 and INPS 2 was significantly different (pairwise comparison: *p*=0.02), and the prognosis of INPS 2, 3, and 4 groups was similar (pairwise comparison: *p*=0.733, 0.742, 0.947). There was no significant difference between INPS 5 and 6 groups (pairwise comparison: *p*=0.827). So INPS was further divided into four groups: patients with INPS 0 were assigned to the Low Risk group(n=106, 20.7%); patients with INPS 1 were assigned to the Low–Medium Risk group(n=145, 28.3%); patients with INPS 2 to 4 were assigned to the High–Medium Risk group(n=209, 40.7%), and patients with INPS 5 to 6 belonged to the High Risk Group(n=53, 10.3%).

### Survival Analysis Based on Individual Biomarkers and INPS Groups

**Figure 3:**
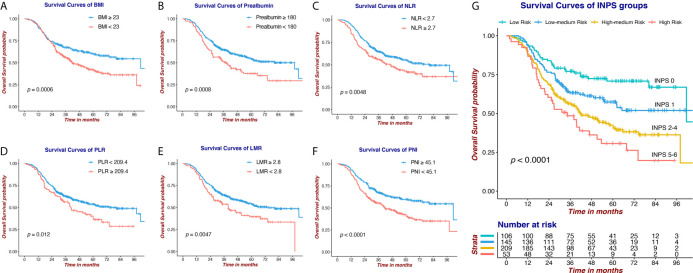
Survival curves *via* Kaplan-Meier analysis of 6 nutritional and Inflammatory biomarkers and INPS groups. **(A)** Survival curves of BMI (≥23 vs <23). **(B)** Survival curves of Prealbumin (≥180 vs <180). **(C)** Survival curves of NLR (<2.7 vs ≥2.7). **(D)** Survival curves of PLR (<209.4 vs ≥209.4). **(E)** Survival curves of LMR (≥2.8 vs <2.8). **(F)** Survival curves of PNI (≥45.1 vs <45.1). **(G)** Survival curves of INPS groups (score 0 vs 1 vs 2-4 vs 5-6). INPS, inflammatory-nutritional prognostic score; BMI, body mass index; NLR, neutrophil-lymphocyte ratio; PLR, platelet-lymphocyte ratio; LMR, lymphocyte-monocyte ratio; PNI, prognostic nutritional index.

Survival curves *via* Kaplan-Meier analyses and log-rank tests of 6 selected nutritional and Inflammatory biomarkers and INPS groups are presented in **Figure 3**. Our results indicated that low BMI, low Prealbumin, high NLR, high PLR, low LMR, and low PNI were significantly associated with shorter OS (HR=1.56, 95% CI:1.21-2.02; HR=1.61, 95% CI:1.21-2.12; HR=1.45, 95% CI:1.12-1.88; HR=1.47, 95% CI:1.09-2.00; HR=1.50, 95% CI:1.13-1.99; HR=1.68, 95% CI:1.30-2.17, respectively). The 5-year OS rates of the patients in the INPS Low, Low–Medium, High–Medium, and High Risk groups were 70.8%, 57.4%, 41.5%, and 30.6%, respectively (Low–Medium Risk vs Low Risk: HR=1.64, 95% CI: 1.06-2.53, *p*=0.026; High–Medium risk vs Low risk: HR = 2.45, 95% CI: 1.65-3.65, *p*<0.001; High risk vs Low risk: HR = 3.30, 95% CI: 2.04-5.35, *p*<0.001).

Univariate analysis and multivariate Cox regression analysis of baseline characteristics and INPS groups are presented in **Table 2**. The potential prognostic factors identified in the univariate analysis (*p ≤* 0.1) were included in the multivariate analysis. According to the multivariate analysis, INPS was an independent survival predictor for stage III GC patients with adjuvant chemotherapy, with the adjusted HR=1.50(0.96-2.32), 2.21(1.46-3.33), and 2.75(1.67-4.55), *p*<0.001. Besides INPS, Gender (*p*=0.015), pT stage (*p*=0.026), pN stage (*p*<0.001), lymphovascular invasion (*p*=0.003) and CEA level (*p*=0.007) were independent prognostic factors for OS.

**Table 2:** Univariate and multivariate analyses of baseline characteristics and INPS groups for overall survival in 513 GC patients.

Patients characteristics	Univariate analysis		Multivariate analysis	
	HR (95%CI)	*p*	HR (95%CI)	*p*
**INPS groups**				
Low-medium risk vs Low risk	1.64 (1.06-2.53)	**0.026**	1.50 (0.96-2.32)	0.073
High-medium risk vs Low risk	2.45 (1.65-3.65)	**<0.001**	2.21 (1.46-3.33)	**<0.001**
High risk vs Low risk	3.30 (2.04-5.35)	**<0.001**	2.75 (1.67-4.55)	**<0.001**
*p* for trend		**<0.001**		**<0.001**
**Gender** (male vs female)	1.34 (1.00-1.78)	**0.047**	1.45 (1.08-1.95)	**0.015**
**Age at diagnosis yr.** (>60 vs ≤60)	1.26 (0.98-1.62)	0.077	1.08 (0.82-1.41)	0.605
**Tumor site**				
Body/angulus vs Cardia/fundus	0.96 (0.67-1.39)	0.842		
Antrum/pylorus vs Cardia/fundus	0.81 (0.57-1.16)	0.251		
**Histological grade** (G3 vs G1/G2)	1.27 (0.97-1.67)	0.085	1.21 (0.91-1.60)	0.190
**Pathological tumor type**				
Mucinous adenocarcinoma or signet-ring cell carcinoma vs Adenocarcinoma ^a^	1.21 (0.85-1.72)	0.299		
**pT stage** (T4 vs T1-3)	1.74 (1.16-2.61)	**0.008**	1.62 (1.06-2.47)	**0.026**
**pN stage**				
N2 vs N1	1.92 (1.09-3.39)	**0.024**	2.12 (1.19-3.78)	**0.011**
N3a vs N1	3.26 (1.93-5.53)	**<0.001**	3.08 (1.80-5.26)	**<0.001**
N3b vs N1	5.27 (3.07-9.06)	**<0.001**	4.17 (2.40-7.25)	**<0.001**
**pTNM stage**				
IIIB vs IIIA	2.37 (1.74-3.22)	**<0.001**		
IIIC vs IIIA	3.66 (2.61-5.13)	**<0.001**		
**Lymphovascular invasion** (yes vs no)	1.89 (1.46-2.44)	**<0.001**	1.52 (1.16-1.99)	**0.003**
**Perineural invasion** (yes vs no)	1.26 (0.98-1.62)	0.078	1.23 (0.94-1.62)	0.135
**Adjuvant chemotherapy**				
Mono- vs combinational chemotherapy	1.45 (1.05-2.01)	**0.023**	1.33 (0.94-1.88)	0.103
**CA12-5** (elevated vs normal)	2.23 (1.53-3.26)	**<0.001**	1.25 (0.83-1.89)	0.286
**CA19-9** (elevated vs normal)	1.59 (1.20-2.10)	**0.001**	1.08 (0.80-1.47)	0.608
**CEA** (elevated vs normal)	1.97 (1.49-2.60)	**<0.001**	1.53 (1.12-2.08)	**0.007**
**AFP** (elevated vs normal)	0.81 (0.46-1.42)	0.461		

a including papillary, tubular, or mixed adenocarcinoma.

GC, gastric cancer; INPS, inflammatory-nutritional prognostic score; CA, carbohydrate antigen; CEA, carcinoembryonic antigen; AFP, alpha fetoprotein; HR, hazard ratio; 95% CI: 95% confidence interval.

Bold values are statistically significant, p < 0.05.

### Construction of a Novel Prognostic Nomogram Based on INPS for Stage III GC Patients

**Figure 4:**
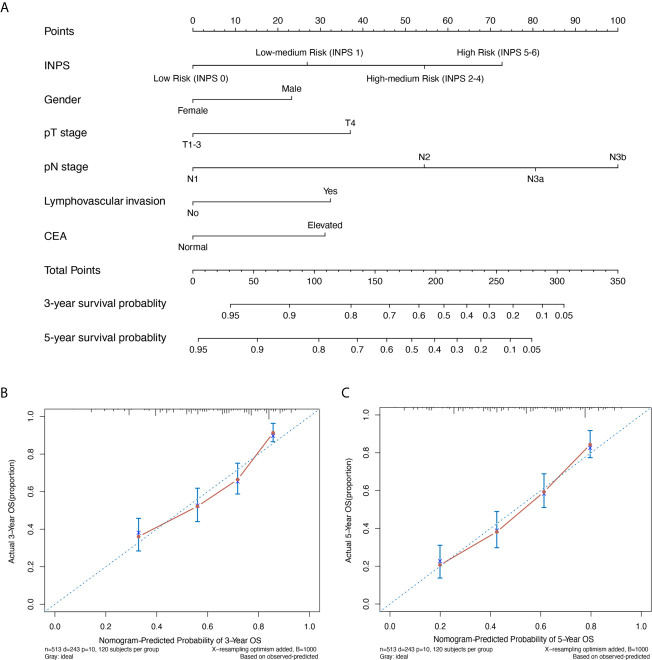
A novel prognostic nomogram based on INPS for stage III GC patients. **(A)** The nomogram for predicting 3- and 5-year survival probability in stage III GC patients. Calibration plots of the nomogram for 3-year **(B)** and 5-year **(C)** survival probability using bootstraps with 1,000 resample. INPS, inflammatory-nutritional prognostic score; GC, gastric cancer; OS, overall survival; CEA, carcinoma embryonic antigen.

A novel prognostic nomogram based on the results of the multivariate analysis using all the independent indicators for OS including INPS, pT stage, pN stage, lymphovascular invasion, and CEA level is presented in **Figure 4A**. A higher total score revealed a worse clinical prognosis of stage III GC patients after surgery. The nomogram showed a good performance with a C-index of 0.707 and an R² of 0.216, which was better than the pTNM stage alone (C-index 0.645, *p*=0.008). The model was internally validated by bootstraps with 1,000 resample with the corrected C-index of 0.693 and the corrected R² of 0.186. Calibration plots of the nomogram (method=‘boot’, B=1000) predicting 3- and 5-year OS also performed well with the ideal model (**Figures 4B, C**).

### Association Between INPS Group and Clinical Characteristics

The relationship between INPS and clinicopathological characteristics is summarized in **Table 3**. The INPS group significantly correlated with the age at diagnosis (*p*<0.001), CA12-5 (*p*=0.005), CA19-9 (*p*=0.008) and AFP (*p*=0.034). There was no significant association between INPS and other clinical characteristics. Our results revealed those patients with older age at diagnosis (>60) and elevated tumor markers (CA19-9, CA12-5, and AFP) had a higher proportion of the INPS High Risk group (score 5-6).

**Table 3:** Association between the INPS group and clinical characteristics.

Clinical characteristics	INPS group (n=513)				*p* value
	Low risk(score 0)	Low-medium risk(score 1)	High-medium risk(score 2-4)	High risk(score 5-6)	
**Gender**					0.107
Male	78(21.9%)	104(29.2%)	133(37.4%)	41(11.5%)	
female	28(17.8%)	41(26.1%)	76(48.4%)	12(7.6%)	
**Age at diagnosis yr.**					**<0.001**
≤ 60	65(24.9%)	79(30.3%)	103(39.5%)	14(5.4%)	
> 60	41(16.3%)	66(26.2%)	106(42.1%)	39(15.5%)	
**Tumor site**					0.714
Cardia/fundus	15(19%)	18(22.8%)	38(48.1%)	8(10.1%)	
Body/angulus	43(23.4%)	54(29.3%)	68(37%)	19(10.3%)	
Antrum/pylorus	48(19.2%)	73(29.2%)	103(41.2%)	26(10.4%)	
**Histological grade**					0.702
G1/G2	32(18.2%)	49(27.8%)	77(43.8%)	18(10.2%)	
G3	74(22%)	96(28.5%)	132(39.2%)	35(10.4%)	
**Pathological tumor type**					0.315
Adenocarcinoma^a^	91(20.5%)	120(27.1%)	183(41.3%)	49(11.1%)	
Mucinous adenocarcinoma or signet-ring cell carcinoma	15(21.4%)	25(35.7%)	26(37.1%)	4(5.7%)	
**pT stage**					0.690
T1-3	17(21%)	27(33.3%)	30(37%)	7(8.6%)	
T4	89(20.6%)	118(27.3%)	179(41.4%)	46(10.6%)	
**pN stage**					0.312
N1	22(28.6%)	16(20.8%)	32(41.6%)	7(9.1%)	
N2	32(25%)	41(32%)	43(33.6%)	12(9.4%)	
N3a	34(17.3%)	56(28.6%)	85(43.4%)	21(10.7%)	
N3b	18(16.1%)	32(28.6%)	49(43.8%)	13(11.6%)	
**pTNM stage**					0.105
IIIA	57(26%)	62(28.3%)	81(37%)	19(8.7%)	
IIIB	37(19.6%)	51(27%)	79(41.8%)	22(11.6%)	
IIIC	12(11.4%)	32(30.5%)	49(46.7%)	12(11.4%)	
**Lymphovascular invasion**					0.570
No	61(22.1%)	81(29.3%)	109(39.5%)	25(9.1%)	
Yes	45(19%)	64(27%)	100(42.2%)	28(11.8%)	
**Perineural invasion**					0.148
No	40(16.7%)	68(28.3%)	103(42.9%)	29(12.1%)	
Yes	66(24.2%)	77(28.2%)	106(38.8%)	24(8.8%)	
**Adjuvant chemotherapy**					0.066
Mono-chemotherapy	9(11.1%)	21(25.9%)	40(49.4%)	11(13.6%)	
Combinational chemotherapy	97(22.5%)	124(28.7%)	169(39.1%)	42(9.7%)	
**CA12-5**					**0.005**
Normal	102(21.7%)	139(29.6%)	184(39.1%)	45(9.6%)	
Elevated	4(9.3%)	6(14%)	25(58.1%)	8(18.6%)	
**CA19-9**					**0.008**
Normal	91(23.2%)	104(26.5%)	163(41.6%)	34(8.7%)	
Elevated	15(12.4%)	41(33.9%)	46(38%)	19(15.7%)	
**CEA**					0.107
Normal	92(23%)	109(27.3%)	159(39.8%)	40(10%)	
Elevated	14(12.4%)	36(31.9%)	50(44.2%)	13(11.5%)	
**AFP**					0.034*
Normal	101(21.1%)	136(28.4%)	198(41.3%)	44(9.2%)	
Elevated	5(14.7%)	9(26.5%)	11(32.4%)	9(26.5%)	

a including papillary, tubular, or mixed adenocarcinoma; *Fisher’s exact test.

INPS, inflammatory-nutritional prognostic score; CA, carbohydrate antigen; CEA, carcinoembryonic antigen; AFP, alpha fetoprotein.

Bold values are statistically significant, p < 0.05.

## Discussion

In China, since the nationwide screening program has not been well developed, locally advanced GC, especially stage III GC, accounts for the majority of resectable GC, resulting in a low overall 5-year survival rate (16, 17). According to the ACTS-GC trial and CLASSIC trial, even after radical gastrectomy followed by standard adjuvant chemotherapy, the prognosis of stage III GC is still very poor (4, 5). Thus, it is quite important to identify the specific biological characteristics for tumor progression so as to make further risk stratification and individualized therapeutic strategy.

The results of the present study indicated that INPS, consisting of preoperative BMI, prealbumin, NLR, PLR, LMR, and PNI, was an independent indicator of outcome in stage III GC patients who underwent surgery followed by adjuvant chemotherapy. Survival analysis showed that INPS could effectively classify patients into four risk groups. Furthermore, we integrated INPS with other independent clinicopathologic predictors to construct a prognostic nomogram for stage III GC patients, which showed a good prognostic performance.

Cancer-related inflammation and malnutrition are quite common in patients with malignant tumors and closely correlated to tumor recurrence and progression (7, 18). In the past few years, a number of studies have explored the prognostic value of some preoperative inflammatory and nutritional biomarkers in GC patients, including NLR, PLR, BMI, PNI, Glasgow Prognostic Score (GPS), and so on, and aimed to find out the most optimal predictor for the outcome. The results were variable and controversial leading to the limited clinical value (8, 11–14, 19). Actually, single indicators have certain limitations, and cannot fully reflect the overall immune and nutritional status of patients. Gennaro Galizia’s study established a new prognostic tool, the Naples prognostic score, including albumin, cholesterol, NLR, and LMR, which showed a better performance than the existing single index in predicting the prognosis of colorectal cancer patients (20). What’s more, most of the studies incorporated these indicators of strong collinearity and correlation into a multivariate cox regression analysis to investigate independent prognostic factors, causing interference between variables and certain statistical problems. Our study included all available parameters as much as possible and used the LASSO Cox regression model to effectively select valuable variables and, to some extent, reduced the influence of multicollinearity.

In our study, BMI, prealbumin, and PNI represented patients’ nutritional status, and NLR, PLR, and LMR represented patients’ immune-inflammatory microenvironment. Interestingly, in line with our previous study and other researches (15, 21), high-BMI patients showed better prognosis, which further confirmed a phenomenon called the “obesity paradox” in the prognosis of GC. Cancer-associated malnutrition also contributes to severe postoperative complications, decreased immunological function, and the activation of the systemic inflammatory response (SIR), leading to poor therapeutic efficacy (7, 22). In addition, elevated neutrophils could create a tumor-favorable microenvironment through secretion of reactive oxygen species (ROS), nitric oxide, and arginase, causing lymphocytes inactivation, while lymphocytes play important roles in immune surveillance and anti-tumor response (23, 24). Monocytes, especially which differentiate into macrophages, play vital roles in cancer development, progression, and metastases (25). And there is accumulating evidence that tumor cell-activated platelets can facilitate cancer survival and dissemination (26). Based on this evidence, the score combining NLR, PLR, and LMR has been proved as an independent prognostic factor in several cancers (10, 27). Some studies identified the immunoscore of tumor tissue and serum interleukin-6 (IL-6), IL-11 or CD4+/CD8+ T cell also reflecting the immunoinflammatory status, but it is hard to be used in clinical practice due to the high cost and inconvenience (28).

In terms of other predictors in the nomogram, consistent with previous researches, pT stage, pN stage, lymphovascular invasion, and elevated CEA, representing the intrinsic characteristics of the tumor, were independent prognostic factors (6, 29). Whether gender is a prognostic factor for GC remains unknown and controversial (30, 31). In our study, men showed worse outcomes than women, probably due to the differences in the age distribution (age>60 was 53.9% in men and 38.2% in women, *p*<0.001), with more chronic diseases in men leading to worse treatment compliance.

The major strengths of this research include the large size of the cohort of postoperative stage III GC, receiving standard D2 gastrectomy by specialized and experienced gastroenterology surgeons at a high-volume comprehensive hospital, where nearly 1,000 GC surgeries are performed per year, and the use of standard adjuvant chemotherapy regimens in all patients. These factors, to some extent, resulted in the consistency between patients and reliable results. Furthermore, this was the first study focusing on stage III GC to comprehensively take into account the intrinsic characteristics of the tumor, the immuno-inflammatory microenvironment, and the nutritional status of the host. More importantly, parameters in INPS are routinely detected, cost-effective, and easily accessible in clinical practice, making it a great and valuable index for prognostic stratification, treatment optimization, and guiding postoperative follow-up strategies. We recommend closer monitoring and more frequent follow-up for High–Medium or High Risk patients to early detect tumor recurrence. Also, various studies have shown that improvement of malnutrition and inflammatory status could lead to fewer postoperative complications and better outcomes (32–34). However, whether it is necessary to add anti-inflammatory drugs or to strengthen chemotherapy regimens for INPS High-risk individuals remains to be further verified in prospective studies.

Our study still has some limitations. It was a retrospective study from a single center, and the results should be further externally validated in multiple health centers or in large-scale prospective cohorts. In addition, due to the specific and complicated biological behavior of cancers, other factors affecting the prognosis of GC (genomics biomarkers, lifestyle habits and socioeconomic status, etc.) were not included in our parameters.

In conclusion, as an available and cost-effective scoring system, preoperative INPS has good clinical application prospects in predicting the postoperative survival of stage III GC patients with adjuvant chemotherapy. The prognostic nomogram based on INPS shows good prognostic performance and may act as an optimal tool for making individualized treatment strategy and follow-up plan.

## Data Availability Statement

The original contributions presented in the study are included in the article/supplementary material. Further inquiries can be directed to the corresponding authors.

## Author Contributions

Conception and design: NW, JZ. Development of methodology: NW, WX, SL, JJ, CW, ZZ, CY. Acquisition of data: NW, WX. Analysis and interpretation of data: NW, WX, ZZ, CY. Writing, review of the manuscript: NW, JL, JZ. Study supervision: JL, JZ. All authors contributed to the article and approved the submitted version.

## Funding

The study was supported by the National Science Foundation of China (81972707).

## Conflict of Interest

The authors declare that the research was conducted in the absence of any commercial or financial relationships that could be construed as a potential conflict of interest.
